# Poly(ADP-Ribose) polymerase1 Has Potential to Facilitate the Nucleosome Disassembly

**DOI:** 10.3390/ijms27156599

**Published:** 2026-07-24

**Authors:** Aleksandr A. Alekseev, Mikhail M. Kutuzov, Ekaterina A. Belousova, Alexander A. Ukraintsev, Ivan D. Goncharov, Aleksandra A. Vasileva, Mikhail A. Khodorkovskii, Olga I. Lavrik

**Affiliations:** 1 Peter the Great St. Petersburg Polytechnic University, Saint Petersburg 195251, Russia; alex35093@gmail.com (A.A.A.); bing_fox.2001@inbox.ru (I.D.G.); avasileva@spbstu.ru (A.A.V.); 2 Institute of Chemical Biology and Fundamental Medicine, Novosibirsk 630090, Russia; kutuzov.mm@mail.ru (M.M.K.); rina@1bio.ru (E.A.B.); alan.ukraintsev@gmail.com (A.A.U.)

**Keywords:** nucleosome, chromatin like particle, poly(ADP-ribose) polymerase1, optical tweezers

## Abstract

Being the basic building blocks of chromatin, nucleosomes and their stability determine the genome accessibility for different DNA-dependent proteins. This characteristic is labile under cell-life processes. One of the abundant DNA-binding proteins, which is important for genome compaction, is poly(ADP-ribose) polymerase1 (PARP1). Despite the extensive experimental data on the chromatin compaction regulation under ADP-ribosylation, the details of the interplay of nucleosome with PARP1 in the absence of protein activation remain unclear. In this study, we discovered unusual changes of the nucleosome wrapping strength upon PARP1 interaction using a single-molecule approach—optical tweezers. We demonstrated that PARP1 binding leads to weakening of the contacts of inner DNA turn in nucleosome.

## 1. Introduction

The chromatin organization is required for DNA compaction as well as the regulation of the DNA-dependent processes [1]. The basic unit of chromatin is the nucleosome core particle (NCP), whose compaction is under fine regulation. There are the following two main mechanisms providing correct DNA compaction: enzymatic, which includes histone posttranslational modifications and the action of ATP-dependent nucleosome remodeling, and the second one is the action of non-histone chromatin proteins changing NCP conformations by binding to them [2].

Poly(ADP-ribose) polymerase1 (PARP1) is best known as a central regulator of DNA damage detection and repair [3] and can affect the NCP structure. It is a clinically validated target, with several approved molecules that are particularly effective in the treatment of cancers bearing different mutations, especially in BRCA genes [4,5,6]. Thus, accurate systematic knowledge about its interaction with genome may lead to suggesting new therapeutic approaches. Multiple studies describe the main PARP1-mediated mechanism of regulation of NCP compaction via histone poly(ADP-ribosyl)ation. At the same time, the binding of PARP1 to the NCP affects nucleosome compaction without activation [7]. Moreover, there are data demonstrating the ability of PARP1 to bind to NCP in a competitive and mutually exclusive manner with a linker histone H1 [8]. It is noteworthy that histone H1 does not influence compaction density of NCP, but it reduces NCP dynamics toward the stabilization of a defined conformation [9,10]. It has been recently shown that PARP1 has a higher affinity to partially unwrapped than to fully wrapped NCP [11]. In this context, a potential influence of PARP1 on the intermediate states of NCP during its dynamics is possible.

In terms of the set of non-covalent bonds dynamically organizing the NCP structure, the existence of several spatial conformations of NCP can be predicted. Moreover, each of them could be characterized by the set of contacts with different total tenacity [12,13,14]. In this work, by using optical tweezers, we estimated the strength of NCP contacts under its binding by PARP1 to disclose the PARP1 potential in chromatin remodeling process.

## 2. Results and Discussion

Optical tweezers are a common method for measuring the intramolecular strength for complex molecules including strength of individual NCP [15]. It should be noted that the average value of NCP unwrapping force can vary in a wide range depending on the sequence of DNA model used [16]. This is mainly based on electrostatic variations of different nucleosome positioning DNA sequences (NPSs) and histone variant sets, as well as the influence of buffer components.

**Figure 1:**
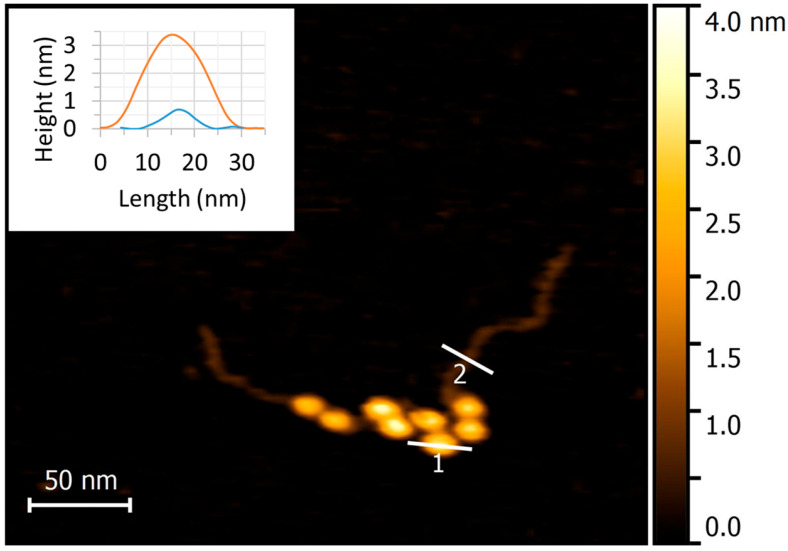
AFM image of reconstituted CLP. The profiles of height and width for bar “1” (in orange) and “2” (in blue) crossing NCP and DNA, correspondingly.

In our work we used a DNA model system based on Widom’s clone 601 with eight tandem NPSs to form chromatin-like particle (CLP). In order to verify our model CLP, we characterized the probe by atomic force microscopy (AFM). The usual AFM image of the reconstituted CLP containing fragment of plasmid is presented in Figure 1 and Figure S1.

Analysis of AFM images confirms the correct NCP reconstitution. Definitely visualized CLP morphologically looks like “beads on a string”. The morphometric parameters of individual NCP correspond to the usual nucleosome size and shape with approximately ~15 nm diameter and ~3.5 nm height (line 1 in Figure 1).

**Figure 2:**
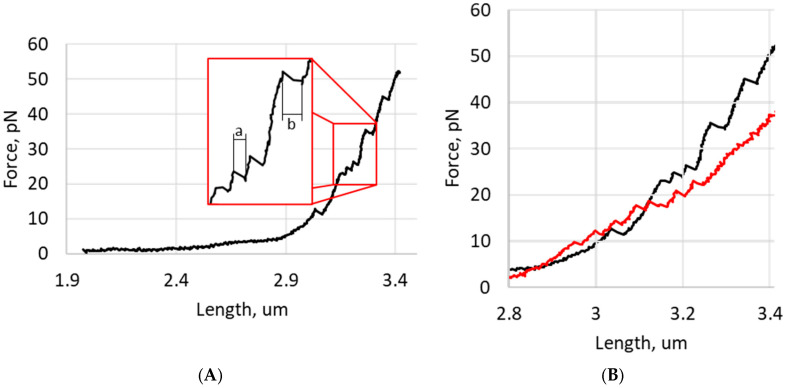
The force-extension profile of NCP unwrapping. Typical force-extension profile demonstrating the dependence of tensile force on NCP-containing DNA probe stretching without PARP1 (black line in (**A**,**B**)) and with 50 nM PARP1 (red line in (**B**)). The characteristic stretching lengths of disassembly of a single (parameter a) and simultaneously two NCPs (parameter b) are 20 and 40 nm, respectively. The primary data for plotting all CLPs force-extension profiles in the presence or absence of PARP1 are presented in File S3.

The typical force-extension profile of the specified probe is in Figure 2A. The profile represents a sequential change of parameters characterized by the intramolecular interaction forces dynamic between parts of the CLP under the molecule tension. It should be noted that regardless of the mono- or polynucleosomal model as well as the DNA sequence used, the minimum probe elongation at unwinding of a single core particle is approximately 40 nm [17]. Therefore, the length changing at the force-extension profile is precisely the parameter by which the particle stability can be controlled.

The observed experimental force-extension profile of the single NCP into the literature exhibited two principal regions [17]. One of those has a slight gradual rise of characteristic rupture force in diapason at approximately 2.55–2.70 μm. It corresponds to the release of the outer DNA turn of the NCP. Another one has “teeth” on the curve and is compliant with the unwrapping of the inner DNA turn from the NCP core.

In Figure 2A(a), the characteristic “teeth” corresponding to the unwrapping of the NCP inner turn led to an elongation of the DNA probe by 20 nm, which corresponds to ~70 base pairs, and are clearly visible in the force range of approximately 10–45 pN. Several “teeth” value unwrapping is about 40 nm, which most likely corresponds to unwrapping of two NCPs simultaneously with the same force value (Figure 2A(b)).

The advantage of Widom’s sequence existence inside the DNA duplex is based on accurate positioning of the histone octamer, forming NCP; however, other sequences would promote nucleosome formation too. Thus, the protocol of CLP reconstitution as an NCP-containing probe does not exclude the possibility of formation of complexes with non-specific sequences that could lead to variation in nucleosome number into each CLP molecule.

**Figure 3:**
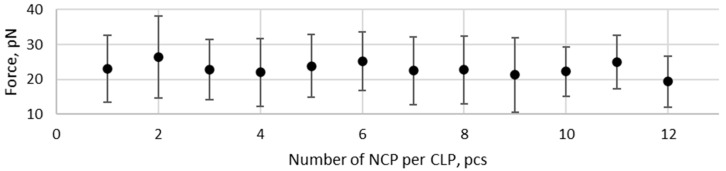
The dispersion of quantifying nucleosome stability against the NCP amount to CLP.

**Figure A1:**
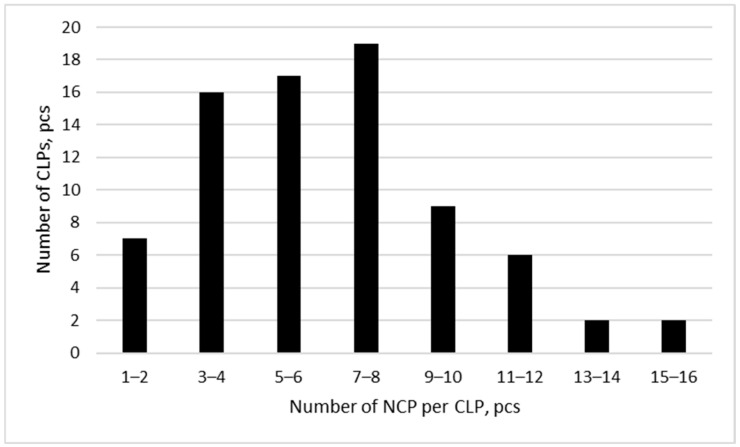
The distribution of the NCP number per CLP. The number of CLP molecules in the calculation is seventy-eight.

It was shown that the extension changes up to 5 pN correspond to the outer half wrap release of a single NCP [17,18,19]. In addition, under unwrapping of the nucleosome using optical tweezers, it is not possible to discriminate between individual unwrapping events of the outer DNA turns with characteristic “teeth”. In our case, changes in the force-extension profiles of more than 5 pN were taken into account only. Thus, given the experimental design, only the inner DNA turn unwrapping events were considered for calculations. The distribution had a maximum of seven–eight NCPs (Figure A1). However, the usage of force-extension profile cannot discriminate against the nucleosomes formed on specific or non-specific sequences. Therefore, all the measured events were processed together. It should be noted that our measurements do not exhibit an essential diversity among the average of individual NCP unwrapping forces calculated for CLP with different amounts of NCPs that lie in the range of 23 pN (Figure 3). It could be a consequence of natural chicken histones used possessing a set of modifications, which potentially follow to produce NCP with alternative stability.

Overall, because the force required to unwrap from one to up to twelve individual core particles as part of CLP is almost the same for all NCPs, we obtained additional support to combine the parameters from all the NCPs for the calculations below.

**Figure 4:**
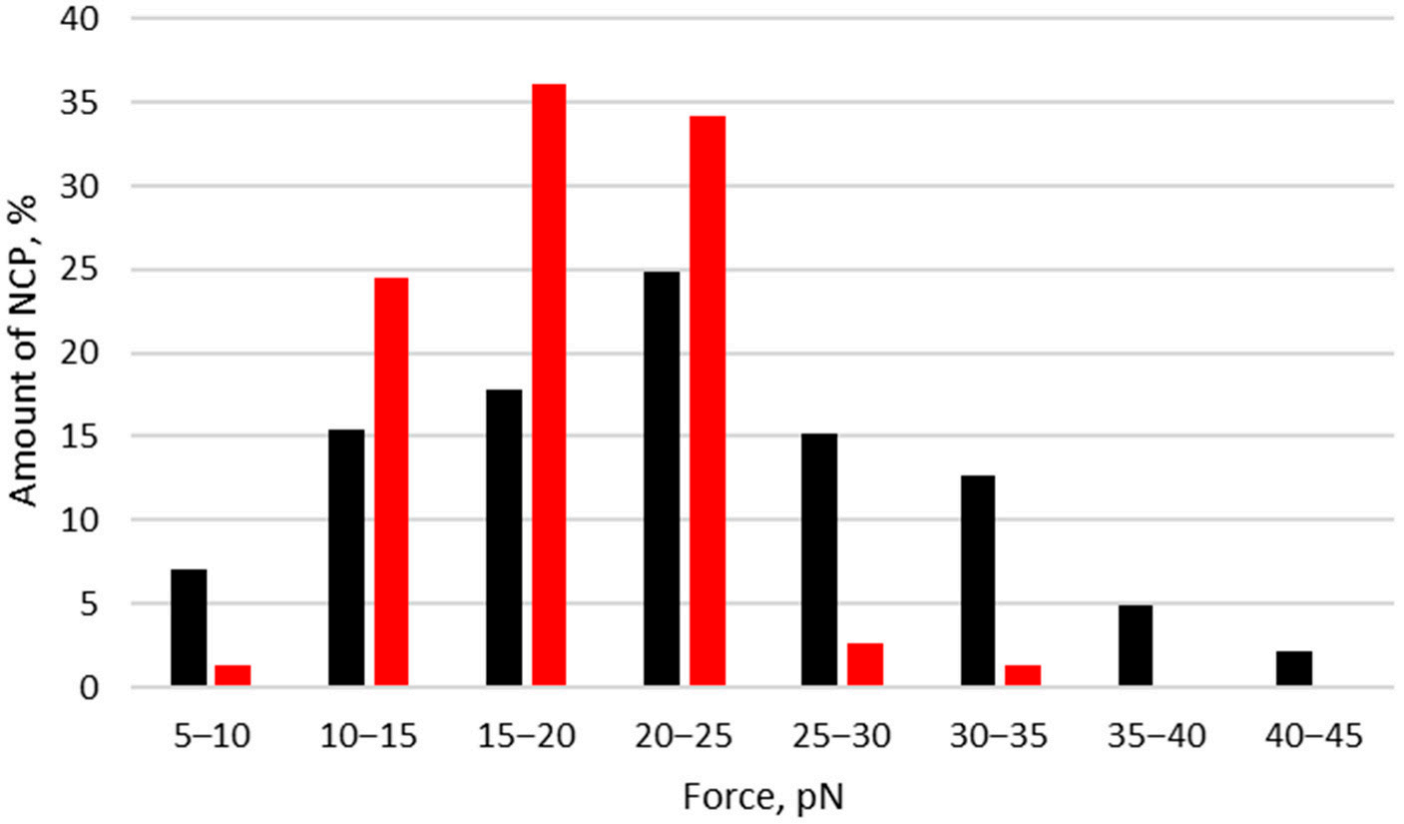
The distribution of NCP unwrapping force. The histograms display the distribution of the amount of NCP complexes by strength tension of probes without PARP1 (black bars) and with PARP1 (red bars). The force ranges of disassembly are on the *X*-axis, in pN. The representation of the NCP complexes at a defined strength range is on the *Y*-axis, in percentage of the total number of nucleosomes in the sampling. The bin width was calculated using the square root rule. The number of NCPs in the calculation is four hundred twenty-seven for CLP without PARP1 and one hundred fifty-five for CLP in presence of PARP1.

Together, these results allow us to combine measurements for calculations. We measured all forces of the CLP force-extension profiles and sorted them into groups with intervals of 5 pN to define the number of NCP per probe molecule. We combined the values of the forces obtained in all stretching experiments and built the distribution of forces that should correspond to NCP amount having a specific contact number (Figure 4).

According to the literature data, the range of forces required for NCP unwrapping could vary in the range of 5–45 pN [18,19]. In our case the majority of NCP unwrapping events in all tensile probes occurred in the range of 15–30 pN (Figure 4).

To define the impact of PARP1 on the NCP tension profile, we performed similar experiments using a freshly assembled CLP with PARP1 (Figure 2B). The typical tension profile in this case demonstrated the difference in characteristic signature of “teeth” (Figure 2B). In particular, these differences were expressed in the lower force magnitude that corresponded to each “tooth” with nearly a total loss of particles that had the highest forces.

The distribution of the NCP unwrapping force in the presence of PARP1 is shown in Figure 4 (bars in red). It is evident that the distribution differs from that shown in Figure 4 (bars in black) by virtual absence of nucleosomes, for which the unwrapping force is 25–45 pN and whose contribution to the distribution in the absence of PARP1 was more than 35%. Altogether, the literature [11,17] and collected results favor the suggestion that PARP1′s interaction with individual NCP may impact NCP stability by destabilizing or altering the quantities of DNA–protein interactions.

**Figure 5:**
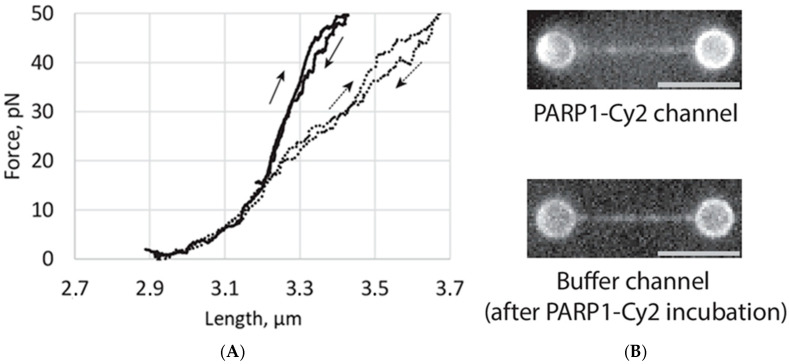
Interaction of PARP1 with DNA. (**A**) Typical force-extension curves of a 9.9 kbp DNA fragment in buffer (solid line) and in the presence of 50 nM PARP1 (dotted line). (**B**) Single-molecule fluorescence imaging of PARP1-Cy2 binding to a representative DNA molecule from the 22–24 kbp preparation at the tension of ~3 pN to keep it extended and prevent potential PARP1-induced DNA condensation. **Top**: DNA in the microfluidic channel containing 50 nM PARP-Cy2; **bottom**: the same DNA molecule after transferring to a buffer channel lacking free PARP1-Cy2. Scale bar, 5 μm. All experimental details can be seen in Materials and Methods. The primary data for plotting all force-extension profiles in the presence or absence of PARP1 are presented in File S6.

It has been shown previously at the sub-piconewton forces diapason that PARP1 binding stabilizes naked DNA compaction [20]. The mechanical peculiarity of the interaction with non-looped DNA strands, however, was not studied. To examine the capability of PARP1 to influence DNA structure, we compared the force-extension curves of naked DNA and its complex with PARP1 (Figure 5). Crucially, prior to complexation, the DNA probe was stretched to a tension of 2–3 pN to exclude loop formation [20]. We observed a noticeable difference in DNA force-extension characteristics at tensile forces greater than 15 pN. The presence of PARP1 led to less steep curve and it permits stretching DNA to a greater length compared to free, naked DNA at the same force values. This phenomenon indicates a decrease in stiffness of DNA duplex and may be likely to implicate the existence of an alternative DNA state at PARP1 interaction. It was shown that zinc finger domains of PARP1 have numerous contacts with both the sugar-phosphate backbone of DNA strands and the edges of base pairs through major and minor grooves, forming a continuous interaction surface on DNA [21]. Moreover, BRCT domain could recognize intact DNA via its positively charged surface [22]. It is possible that these interactions lead to deformation of the B-duplex, which is reflected in a decrease in the rigidity of the DNA duplex observed in our experiment. It could be an additional argument of the multifaceted interaction of PARP1 with a different part of the genome.

There are abundant data about the influence of PARP1 on the NCP state in the context of genome compaction. The recent studies even proposed the complex model of interaction of PARP1 with NCP in the linker region [23,24]. The smFRET-based studies disclosed distance changes between neighboring gyres of nucleosomal DNA in the axial direction under PARP1 binding that could lead to distortion of the canonical NCP conformation [7]. In spite of that, our AFM study of the NCP compaction displays no significant changes of DNA arms positioning in the radial direction upon PARP1 binding [25]. Contradictory results could be a consequence of the approach’s limitations: the AFM technique suggests molecular adsorption on the mica surface and could be a failure to detect the axial distortions of DNA into NCP. Thus, the results obtained by the smFRET and AFM assays could be considered as supplementing each other.

Additionally, PARP1 has recently been shown to demonstrate higher affinity to partially unwrapped NCP over the canonical one [11]. Moreover, intriguing results allow to hypothesize the competition between PARP1 and linker histone H1 for NCP binding [23,26,27,28]. The interplay between the functioning of PARP1 and chromatin remodeling factors was shown. For example, ATP-dependent factor ALC-1 exhibits the cooperative action to PARP1 on NCP under PARP1 activation [29]. Further, the details remain to be determined, but there is some evidence that PARP1 and its interaction with the nucleosome core particle play a role in ALC1-dependent nucleosome remodeling [30]. Thus, altogether with our results, it may suggest a potential role of PARP1 in the reorganization of NCP in a deeper stage when the NCP is not already fully wrapped with DNA.

## 3. Materials and Methods

### 3.1. DNA Constructs and Proteins

Human PARP1 and chicken histones are purified as described earlier [31,32]. The 9.9 kbp fragment bearing eight tandem Widom’s 601 sequences separated by 25 bp linkers was released by XbaI (New England Biolabs, Ipswich, MA, USA) digestion from the pKYB1-based plasmid containing the corresponding 8 × NPS inserts [33]. The linearized plasmid was then ligated with a 50-fold molar excess of biotinylated oligonucleotide (5′-CTAGCGAGTGXXXXX-3′; X denotes biotin tag) in the presence of a short helper oligonucleotide (5′-CACTCG-3′) to facilitate efficient ligation to the XbaI overhang [34]. DNA primers and oligonucleotides for DNA synthesis were obtained at Lumiprobe RUS Ltd. (Moscow, Russia) and at the Laboratory of Biomedical Chemistry (Institute of Chemical Biology and Fundamental Medicine, Siberian Branch of the Russian Academy of Sciences, Russia) Unincorporated oligonucleotides were removed by gel filtration through a Bio-Spin P-30 column (Bio-Rad, Hercules, CA, USA). Nucleosomes were then reconstituted onto the resulting biotinylated DNA construct, as described in [32], with minor variations. Certainly, here we do not perform the pre-reconstitution of NCP and verify the efficiency of NCP preparation by AFM and optical tweezers instead of native gel electrophoresis.

For single-molecule fluorescence experiments, a set of linear DNA molecules of 22 kbp and 24 kbp was prepared from bacteriophage λ DNA (48.5 kbp) as previously described [35]. The DNA was nucleosome-free, double-stranded, and contained no intentionally introduced damage.

Fluorescent labelling of PARP1 on the terminal amino group using succinimidyl esters of 5(6)-carboxycyanine2 (Cy2-SE) was performed as described [36]. The stoichiometry of protein labelling did not exceed 1 mole of dye per mole of protein.

### 3.2. Atomic Force Experiments

For AFM experiments, NCP assembly was performed using a DNA fragment containing eight NPSs. For this purpose, the corresponding DNA fragment was amplified using PCR. Then, the reaction mixture was purified in 0.7% agarose gel under native conditions. The corresponding bands with amplicon were visualized with transilluminator, and cut out, followed by purification with cleanup kit «Cleanup Standart» (Evrogen, Moscow, Russia). NCP reconstitution was provided as described in [32] with minor variations. Certainly, we do not perform the pre-reconstitution of NCP and verify the efficiency of CLP preparation by AFM instead of native gel electrophoresis. To that end, the samples were diluted with buffer solution containing 50 mM HEPES (pH = 8.0) and 5 mM NiCl2 to final concentration of 1 nM and immediately applied to the freshly cleaved mica surface. After application, the mica surface was rinsed three times with 1 mL of clean Milli-Q water and dried under a gentle stream of argon. The samples were stored in a desiccator before imaging. Scanning was performed on a “Multimode 8” in tapping mode in air. The carbon cantilever NSG01_DLC (NT-MDT, Zelenograd, Russia) with a tip-resonant frequency of 90–180 kHz was used to scan CLP. A typical resulting image had a size of 2 μm × 2 μm at 1024 pixels per line, 4 μm × 4 μm at 2048 pixels per line, or 8 μm × 8 μm at 4096 pixels per line. The scanning frequency was 1.0, 0.5 or 0.25 Hz, respectively.

### 3.3. Optical Tweezer Setup

Optical trapping experiments were performed using a custom dual-trap instrument built around a 1064 nm Nd:YVO_4_ CW laser (5 W, BL-106C, Spectra-Physics, Milpitas, CA, USA) and a high-NA oil-immersion objective (LOMO 100×, NA 1.25, LOMO, St. Petersburg, Russia), essentially as detailed previously [35,37]. One trap was steered with sub-nanometer precision using a piezo-driven mirror (S-330.80L, Physik Instrumente, Karlsruhe, Germany). Trap stiffness was determined by the viscous-drag method on a high-precision piezo stage (P-561.3DD, Physik Instrumente, Karlsruhe, Germany). Real-time force and bead-to-bead distance were recorded at 33 Hz via bead-tracking in LabVIEW. Measurements were carried out in a five-channel microfluidic chamber (Lumicks, Amsterdam, The Netherlands), consisting of five channels without physical boundaries and separated by laminar flow of a different buffer only. In this case, mixing the substrates from different channels is possible exclusively through diffusion and is prevented by adjusting the high flow rates. This approach allows the transfer of a tethered DNA molecule between channels within the chamber.

### 3.4. Single-Molecule Assay

All single-molecule experiments were performed at 22 °C in a buffer containing 20 mM Hepes-NaOH (pH 7.5), 100 mM NaCl, 2 mM MgCl_2_, 0.2% (*w*/*v*) BSA, and 0.02% (*v*/*v*) Tween-20. Microfluidic channels were blocked with 0.5% (*w*/*v*) Pluronic F-127 and 0.1% (*w*/*v*) BSA [38]. Most reagents used in the work were manufactured by Sigma (Burlington, MA, USA); bromophenol blue and xylene cyanol were manufactured by Fluka (Merk, Darmstadt, Germany). One of the five channels of the microfluidic chamber was filled with 0.01% (*w*/*v*) 2.1 μm streptavidin-coated polystyrene beads (Spherotech, Lake Forest, IL, USA), and the neighbouring channel contained 50 pM probes in measurement buffer. Two beads were optically trapped in the bead channel, moved into the DNA channel, and a single probe molecule was tethered between them. Successful tethering and the presence of multiple nucleosomes were confirmed by the characteristic-force rip pattern. Force-extension curves were recorded in position-clamp mode by increasing the distance between the traps at 0.1 μm s^−1^ while monitoring bead positions and force with 30 ms time resolution.

When the impact of PARP1 on NCP was estimated, the probe tether adjusted to a tension of 2–3 pN, and keeping the trap positions fixed, the tethered probe was rapidly moved into the adjacent channel containing 50 nM PARP1 in measurement buffer. After 1 min incubation at the tension of 2–3 pN, the force-extension curve was recorded in the channel with PARP1.

Single-molecule fluorescence imaging of PARP1-Cy2 binding to trapped DNA molecules was performed using essentially the same acquisition settings and optical configuration as previously described [35]. Briefly, fluorescence was excited with a 473 nm continuous-wave laser (100 mW), attenuated using a 2.0 OD neutral density filter. Images were acquired with an EMCCD camera (Azimuth Photonics, Moscow, Russia) at 100 ms exposure time and an electron multiplier gain of 3000 and stored as uncompressed TIFF files. During imaging, the DNA was maintained under mild tension of ~3 pN to keep it extended and prevent potential PARP1-induced DNA condensation. The incubation time with PARP1-Cy2 was 1 min. Images were processed in ImageJ (v1.54g). Background illumination gradients were corrected by local background subtraction. Brightness and contrast were adjusted linearly. Unprocessed (raw) images are provided in Files S4 and S5.

### 3.5. Calculations

All the measured forces of CLP disassembly curves were sorted into groups with force intervals of 5 pN. The bin width is calculated using the square root rule. The histograms are the representation of particles in each group as a percentage of the total amount of the acquired particles. The total amount of the calculated experimental force-extension curves was 72 for CLP tension and 23 for tension of the CLP after incubation with PARP1. Primary data are presented at File S3.

## 4. Conclusions

In this study, by using optical tweezers, we demonstrated the changes in the nucleosome core wrapping tension in the presence of PARP1. Overall, the available data suggest that the PARP1 interaction with the nucleosome is complicated. Outside the context of DNA damage, PARP1 may act as a regulator of nucleosome core density both directly and by interaction of PARP1 with linker DNA regions. Alternatively, PARP1 may interact directly with nucleosomal DNA and it reduces the energy barrier at the loosening or unwrapping of the nucleosome core. This potential mechanism agrees well with the monkey bar model proposed earlier for the interaction of PARP1 with DNA [39].

Moreover, it is possible that the site of PARP1 binding affects not only the density of the nucleosome core itself but also influences the recruitment of specific proteins that mediate nucleosome remodeling or sliding. Since nucleosomes undergo remodeling under DNA damage response to implement an alternative gene expression, PARP1 could be one of the regulators of stress-susceptible or stress-resilient factors in the implementation of DNA-dependent processes.

## Data Availability

The original contributions presented in this study are included in the article/Supplementary Material. Further inquiries can be directed to the corresponding authors.

## Supplementary Materials

The following supporting information can be downloaded at https://www.mdpi.com/article/10.3390/ijms27156599/s1.

## Abbreviations

The following abbreviations are used in this manuscript:

AFM	Atomic force microscopy
CLP	Chromatin-like particle
H1	Histone1
NCP	Nucleosome core particle
NPS	Nucleosome positioning DNA sequences
PARP1	Poly(ADP-ribose) polymerase1
