# Supplementary material for: Poly(ADP-Ribose) polymerase1 Has Potential to Facilitate the Nucleosome Disassembly
Source: Int J Mol Sci. 2026 Jul 24;27(15):6599. doi: 10.3390/ijms27156599 (PMC13465022; doi:10.3390/ijms27156599)
Supplement: Supplementary file 1 [file ijms-27-06599-s001.zip › File S1.pdf]

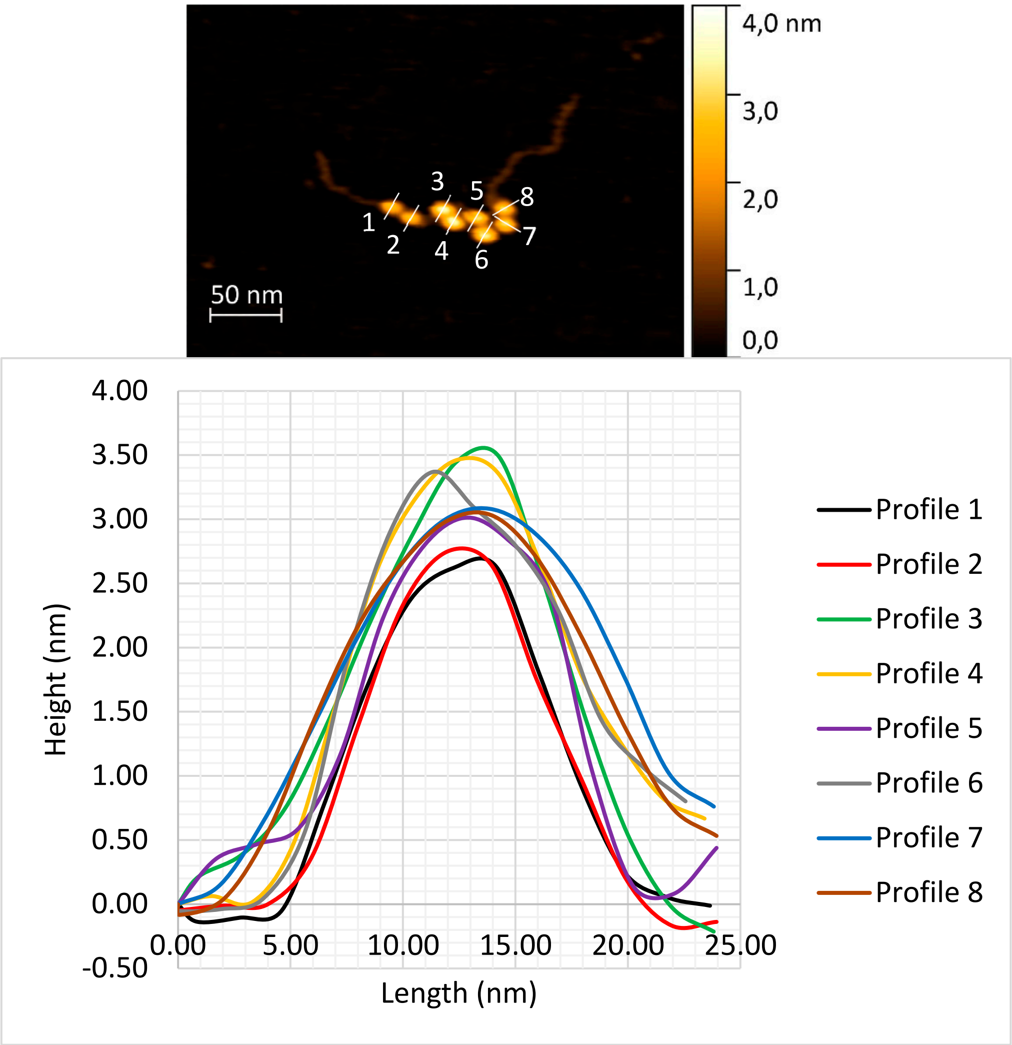

Figure S1. Typical AFM image presented reconstituted CLP from eight NCP (upper part). The profiles of each NCP height are presented in the lower part.
